# Exosomal miR-590-5p in Serum as a Biomarker for the Diagnosis and Prognosis of Gastric Cancer

**DOI:** 10.3389/fmolb.2021.636566

**Published:** 2021-02-12

**Authors:** Guo-Dian Zheng, Zhi-Yuan Xu, Can Hu, Hang Lv, Hua-Xia Xie, Ting Huang, Yan-Qiang Zhang, Gui-Ping Chen, Yu-Fei Fu, Xiang-Dong Cheng

**Affiliations:** ^1^ Department of Hepatobiliary Surgery, Zhejiang Provincial Hospital of Traditional Chinese Medicine, Hangzhou, China; ^2^ Department of Gastric Surgery, Institute of Cancer Research and Basic Medical Sciences of Chinese Academy of Sciences, Zhejiang Cancer Hospital, Cancer Hospital of University of Chinese Academy of Sciences, Hangzhou, China; ^3^ The 2nd Clinical Medical College, Zhejiang Chinese Medical University, Hangzhou, China; ^4^ Laboratory of Digestive Pathophysiology of Zhejiang Province, Institute of Cancer Research, First Affiliated Hospital, Zhejiang Chinese Medical University, Hangzhou, China; ^5^ Department of General Surgery, Zhejiang Provincial Hospital of Traditional Chinese Medicine, Hangzhou, China; ^6^ Department of Gastroenterological Surgery, Zhejiang Integrated Traditional and Western Medicine Hospital, Hangzhou, China

**Keywords:** exosome, miRNA-590-5p, gastric cancer, serum, biomarker

## Abstract

The purpose of this study is to explore the expression of miRNA-590-5p, an exosome of gastric cancer (GC), and to evaluate the suitability of miR-590-5p, an exosome with its own clinical characteristics. Serum samples from 168 gastric cancer patients and 50 matched controls were collected and exosomal RNAs were extracted. After that, miR-590-5p is analyzed by quantitative polymerase chain reaction (qRT-PCR), which is more related to clinical and pathological parameters and patient monitoring data. MGC-803 and HGC-27 cells were treated by miR-590-5p mimics, and then the changes of cell fluidity and invasiveness were monitored. The results showed that the expression level of miR-590-5p in exosomes of healthy observation group, early (I and II) stage group, and late stage (III) group was 30.34 ± 6.35, 6.19 ± 0.81, and 2.9 ± 0.19, respectively (all *p* < 0.05). ROC (receiver-operating characteristic curve) showed that the AUC (area under the curve) of exosomal miR-590-5p was 0.810 with 63.7% sensitivity and 86% specificity. The expression of exosomal miR-590-5p in serum was related to clinical stage (*p* = 0.008), infiltration depth, and the expression level of ki-67 (*p* < 0.001). In addition, Kaplan-Meier analysis showed that the decrease of explicit level of exosomal miR-590-5p was related to the decrease of overall survival rate (*p* < 0.001). Cox regression analysis showed that miR-590-5p can be used as an independent predictor. Furthermore, upregulation of miR-590-5p inhibited cell migration and invasion in MGC-803 cells and HGC-27 cells. The serum expression level of exosomal miR-590-5p may be a biomarker, which is potentially useful and noninvasive for early detection and prediction of GC. In addition, miR-590-5p can play a role in eliminating carcinogens by actively regulating the malignant potential of gastric cancer.

## Introduction

The fourth most common malignant tumor in the world is gastric cancer (GC). GC is still the third leading cause of cancer-related death in the world, because the early diagnosis of gastric cancer is stagnant, there is no improvement, and there is no ideal treatment strategy (Ferlay et al., 2015). Increasing the accuracy of detection of gastric cancer biomarkers can reduce their mortality. Although the development of new biomarkers in blood tests has shown great potential, the development of clinical validation of effective cancer detection markers remains a challenge for a variety of human cancers (Iorio and Croce, 2009). Therefore, looking for a more accurate representation of GC biology features and better diagnostic biomarkers is very important and valuable for the screening of early GC in addition to predicting clinical outcomes, which will allow more patients to receive curative surgery.

MicroRNA (miRNA) is a short-chain noncoding RNA molecule (about 22 nucleotides in length). It regulates protein expression of a particular mRNA by incomplete base pairing, causing inhibition of protein translation of the target gene (Bartel, 2004; Carthew and Sontheimer, 2009). Because miRNAs play a role in carcinogenesis or tumor inhibition during tumor development, research on it has been extended to many types of tumors. Recent studies have shown that abnormal expression of mature miRNAs may be helpful for early detection of gastric cancer (Chen et al., 2014; Fu et al., 2014; Zhang et al., 2015). For example, Rui Liu et al. identified 21 miRNAs which were found differentially expressed in GC. Five kinds of serum miRNAs (miR-20a, miR-34, miR-27a, miR-423-5p, and miR-1) were compared with the control group (Liu et al., 2011).

It is actively secreted by various living cells, and foreign bodies are a group of vesicles of size 50–150 nm. They have physiological functions including immune modulation (Pan et al., 1985; Gross et al., 2012). In the process of inward budding of endosomes in late stage, they develop into intracellular multivesicular endosomes. Besides that, exosomes nucleic acids and proteins exist in exosomes (Trajkovic et al., 2008; Demory Beckler et al., 2013), thereby acting as essential medium for intercellular communication (Bang and Thum, 2012; Waldenstrom and Ronquist, 2014). Separating and identifying specific foreign substances of cancer in body fluids, and subsequently identifying DNA, nucleic acids, and proteins without foreign noncancer pollution, can contribute to the diagnosis and treatment of cancer. It has been found that exosomes prevent their miRNAs from being degraded by RNase (Koga et al., 2011) and remain stable for 5 years at minus 20°C, even after 2 weeks at 4°C. Moreover, they are resistant to freeze-thaw cycles (Weber et al., 2010). Because of its ease of access and stability, exosomal miRNA is considered as a new and slightly invasive cancer diagnosis tool, which may have precalculated value. MiR-590-5p can play a role in carcinogen or tumor inhibitor of vulvar cancer and rectal cancer (Zhou et al., 2016a; Yang and Wu, 2016). However, the relationship between the expression of exosomal miR-590-5p and the clinical features of gastric cancer has not been reported, and the potential correlation between the expression of exosomal miR-590-5p and the treatment and prognosis of gastric cancer needs further study.

The purpose of this study is to explore the clinical significance of serum miR-590-5p and its role in metastasis and invasion of infectious cancer cells. Our current research results show that miR-590-5p can limit the spread and invasion of GC cells, so it may be a potential biological indicator of GC cells being attacked.

## Patients and Methods

### Clinical Samples

168 patients with gastric cancer who visited Zhejiang Cancer Hospital from March 2008 to November 2011 were included in the gastric cancer group. The average age of 168 patients with gastric cancer was 61 years (31–86 years). All patients with gastric cancer were confirmed by histopathology, and the tumor stage was determined according to the lymph node metastasis system in union for international cancer control. Patients who suffered from other cancers were excluded from this study. A healthy control group of 50 volunteers, who visited the hospital for physical examination were enrolled; the average age was 40 years (26–59 years). Volunteers were diagnosed through internal inspection and on-site inspection. None of the patients had received chemotherapy, radiation, or other preoperative tumor treatments. Serum samples were centrifuged for 10 min at 3,000 rpm and then stored at -80°C.

### Follow-up

The survival time of all patients was calculated from the date of diagnosis to the deadline for follow-up which was December 31, 2016. The follow-up period was (5.35 ± 1.52) years, and the median follow-up was 5.35 (3.61–7.67) years. During the follow-up period, 38 patients had recurrence and metastasis, where 33 cases died of GC.

### Isolation of Exosomes From Serum

All of the frozen serum samples were thawed in a 25°C water bath until they were completely liquid and placed on ice until needed. Then the serum sample was centrifuged at 2000 ×g for 30 min to remove cells and debris. The upper liquid containing clear serum was transferred to a new test tube without affecting precipitation and placed in ice until it is ready for separation. Next, the required volume of clarified serum was transferred to a new tube and 0.2 volumes of the Total Exosome Isolation (from serum) reagent (cat no. 4478360; Invitrogen; Thermo Fisher Scientific, Inc.) was added; at this point the solution was thoroughly mixed by pipetting up and down until made homogenous. Samples were incubated for 30 min at an ambient temperature of 4°C and then at an ambient temperature of 10,000 ×g for 10 min. The waste liquid was inhaled and discarded, and the exosome pellet was resuspended in 1 × PBS and subsequently stored for a short time at 4°C.

### Transmission Electron Microscopy

The 400-mesh carbon coated grid was placed to float on a droplet sample for 15 s. Then, we use clean filter paper to move the grid and drain excess liquid from the edge of the grid. The grid was exposed to a drop of 2% of uranyl or phosphotungstic acid at pH 7.0 for about 8 s and excess liquid was drained off. The mosquito net was dried for 8 min. Samples were monitored under 80 kV using an JEC-1200EX microscope (Akasaka Province, Japan).

### Western Blot Analysis

Pellets of exosomes were collected and dissolved in SDS-based buffer. Proteins were quantified by tetracyclic protein detection kit (Beijing Genetically Modified Organisms Technology Co., Ltd., Beijing, China). Protein samples were separated by SDS-PAGE and then transferred to a membrane containing polyvinylidene fluoride (primo bo, Massachusetts, USA). After 1 h, the membrane was separated from the selected first antigen body with 5% skim milk: 1:1,500 diluted anti-CD9 (Abcam, #ab92726) and anti-CD63 (Abcam, #ab134045) overnight at 4°C. Next, they were probed with corresponding secondary antibody conjugated to horseradish peroxidase for 2 h at room temperature. An ECL kit (Millipore) was used to reveal the immunoblots.

### Exosome Quantification and Purity Assessment

The intensity, volume, and distribution of exosomes were analyzed by dynamic light scattering (DLS). Exosomes were suspended in 1× PBS and analyzed with the DLS instrument of Zetasizer Nano ZS (Malvern Instruments Ltd., Worcestershire, UK). All the data were collected and repeated at least three times.

### RNA Extraction From Exosome and qRT-PCR

Total RNAs including miRNAs were extracted from serum exosome using Total Exosome RNA and Protein Isolation Kit (inversion # 4478545). According to the manufacturer's instructions, c-DNA synthesis was performed using the miScript II RT kit (Qiagen, # 218161) according to the manufacturer’s instructions. Quantitative real-time PCR (qRT-PCR) was performed using the miScript SYBR green PCR kit (Qiagen, # 218075). The use of a customized miScript miRNA PCR array in a 384-well array produces the miR-590-5p expression profile (Qiagen, #CM1HS0064C), manufacturer's instructions: use ABI 7900 high-temperature real-time rapid polymerization chain reaction system, circulating at 95°C for 15 min, circulating at 94°C for 15 s, circulating at 55°C for 30 s and circulating at 70°C for 40 cycles. The threshold period data were analyzed by SDS software. And the data is standardized as ce-miR-39 (Takara Bio Company, Tokyo, Japan), a synthetic nonhuman miRNA; at the beginning of RNA isolation, in order to normalize the size of serum and determine whether our miRNA analysis by quantitative polymerase chain reaction (PCR) belongs to linear test range, ce-miR-39 will be referred to in polybrominated biphenyl insulating solution before RNA extraction. A total of 3.5 ul was added to each sample. The relative expression level of miR-590-5p was normalized to ce-miR-39, and the fold change of miR-590-5p expression relative to healthy control group was analyzed by 2^−ΔΔCT^ method. ΔC_T_ and ΔC_T_ and ΔΔC_T_ are calculated using the following formula:

$$\begin{array}{l} \Delta {\text{C}}_{\text{T}}={\text{C}}_{\text{T}\;\text{sample}}-{\text{C}}_{\text{T}\;\text{ce}-\text{miR}-39},\\ \Delta \Delta {\text{C}}_{\text{T}}=\Delta {\text{C}}_{\text{T}\;\text{case}}-\Delta {\text{C}}_{\text{T}\;\text{control}}. \end{array}$$

### Cell Culture

Among the cancer cells listed in MGC-803 and HGC-27, there are cells from Chinese Academy of Sciences. These cells grow in an environment with a carbon dioxide content of 5% at a high temperature of 37°C, and 10% of active bovine serum is added in these environments, in 25 ml culture flasks.

### Transfection of miR-590-5p Mimics

On the day before the transformation, MGC-803 and HGC-27 were vaccinated on six wells to ensure that 70% of the cells were integrated during the transformation. MiR-590-5p mimics were purchased from Biomics (Jiangsu, China); according to the manufacturer's instructions, Lipofectamine 2000 (Invitrogen) was used for redyeing. Oligonucleotides were used when the final concentration was 100 nM. For migration and invasion, cells were collected within 24 h after infection. As controls, all cell lines were used in regular culture conditions, incubated with Lipofectamine 2000 (Mock) or negative control (Control).

### Scratch-Wound Assay

In 160 μL DMEM medium, cancer cells were inoculated with 4 × 10^5^ cells/ml vaccine, and two wells (same as above, Munich, Germany) were cultured for 16 h until polymerization was achieved. After the culture was removed, the cells were washed twice with PBS and closed in DMEM containing 1% fetal bovine serum for 24 h. For each wound, blank area at specific time points after migration was measured. All healing tests were conducted three times and repeated at least five times. The closing rate is calculated using the following formula: closing rate (%) = (initial empty space - empty space after migration)/initial empty space × 100%.

### Cell Invasion Assay

100 μL of cell suspension (2×10^4^ cells) was added to the top chamber of a 24-well plate with 8 μm pores’ membrane (Corning Incorporated, Corning, NY, USA) coated with Matrigel (BD Biosciences, Franklin Lakes, NJ, USA). The lower cavity is filled with 500 microliters of DME containing 20% fetal bovine serum and small bubbles. After incubation in the incubator for 24 h, the filter film was stirred into the methanol for 30 min and dyed in crystallized purple for 10 min. Number of cells attached to the lower surface of a polycarbonate film was counted using a high-speed microscope (×100) and averaged by using five random fields.

### Statistical Analysis

The significance of exosomal miRNA-590-5p expression level difference between patients with gastric cancer and the heathy control was analyzed using nonparametric Mann-Whitney U test. The relationship between exosomal miRNA-590-5p expression and clinicopathological features was assessed using χ^2^ test. Analysis of the ROC, the Kaplan-Meyer survival analysis, and Cox proportional risk model were performed using SPSS (version 24.0) and prism 8 (GraphPad software). The model was applied to the multivariate analysis to determine the independent survival forecast factor, with the *p* value being double and the *p* < 0.05 being considered significant.

## Results

### Identification and Characterization of Exosomes

**FIGURE 1:**
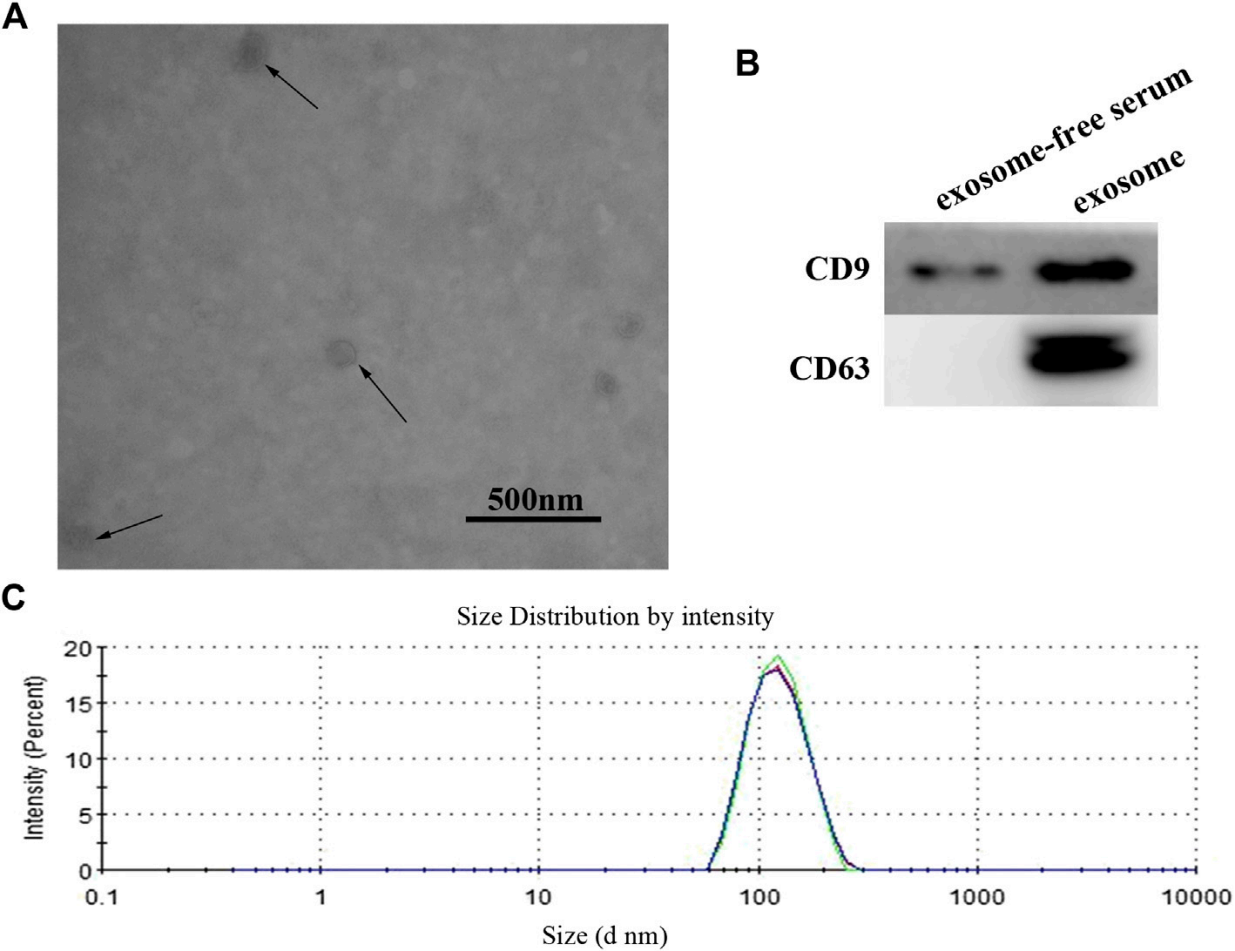
**(A)** Identification and characterization of exosome. The purified exosome from serum of GC patients was observed under a transmission electron microscope (scale 500 nm). **(B)** Western blot analysis of CD9 and CD63 expression in free-exosome serum and exosomes isolated from GC serum. **(C)** The size of exosomes was determined by using the DLS analysis.

In order to verify the efficacy of serum exosomes separation, we analyzed the characteristics of exosomes by using TEM (transmission electron microscopy) and Western blotting. The serum exosomes showed a circular vesicle with a diameter of about 100 nm (Figure 1A). The exosome markers, CD9 and CD63, could be detected in isolated exosomes (Figure 1B). The quality of exosome preparation was further verified using dynamic light scattering (DLS), as shown in Figure 1C. Our results confirmed successful isolation of exosomes from serum samples.

### Serum Exosomal miR-590-5p Expression Was Significantly Lower in Gastric Cancer Patients

**FIGURE 2:**
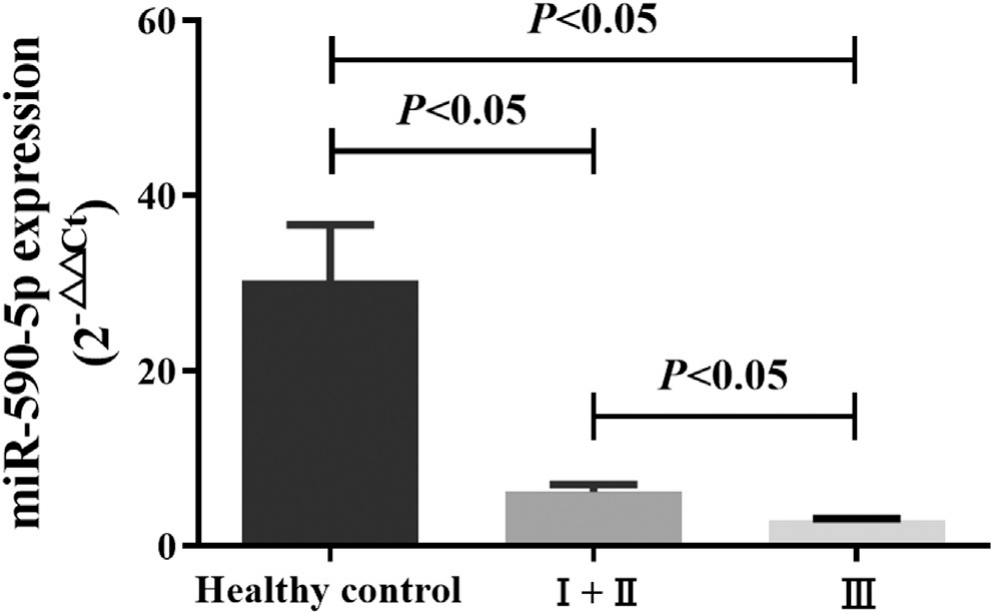
Comparison of serum exosomal miR-590-5p in GC patients and healthy controls. Expression of serum exosomal miR-590-5p was determined by qRT-PCR in 50 healthy controls and 168 GC patients (36 patients in early stages (I and II) and 132 patients in late stages (III)). Ce-miR-39 is used as an internal reference. All data shown were the means ± SEM. *p* < 0.05.

The level of expression of miR-590-5p in the exosome was assessed on 168 patients with GC and 50 healthy controls. Expression level of the exosome of the healthy control group miR-590-5p, early (I and II) stage group, and late stage (III) group was 30.34 ± 6.35, 6.19 ± 0.81, and 2.9 ± 0.19, respectively. The exosomal miR-590-5p expression levels (relative expression normalized by ce-miR-39) were significantly decreased in GC patients compared to the healthy controls (0.12-fold, *p* < 0.05). Moreover, we found that the level of expression of the exosomal miR-590-5p of the gastric cancer group was significantly lower than that of the healthy control group, in both the early and late stages of GC (0.20-fold, *p* = 0.0063, and 0.10-fold, *p* < 0.05, respectively; Figure 2). Of importance, the levels of exosomal miR-590-5p were significantly lower in the late stages than in early stage (0.47-fold, *p* < 0.05).

### Diagnostic Value of Exosomal miR-590-5p in Peripheral Serum

**FIGURE 3:**
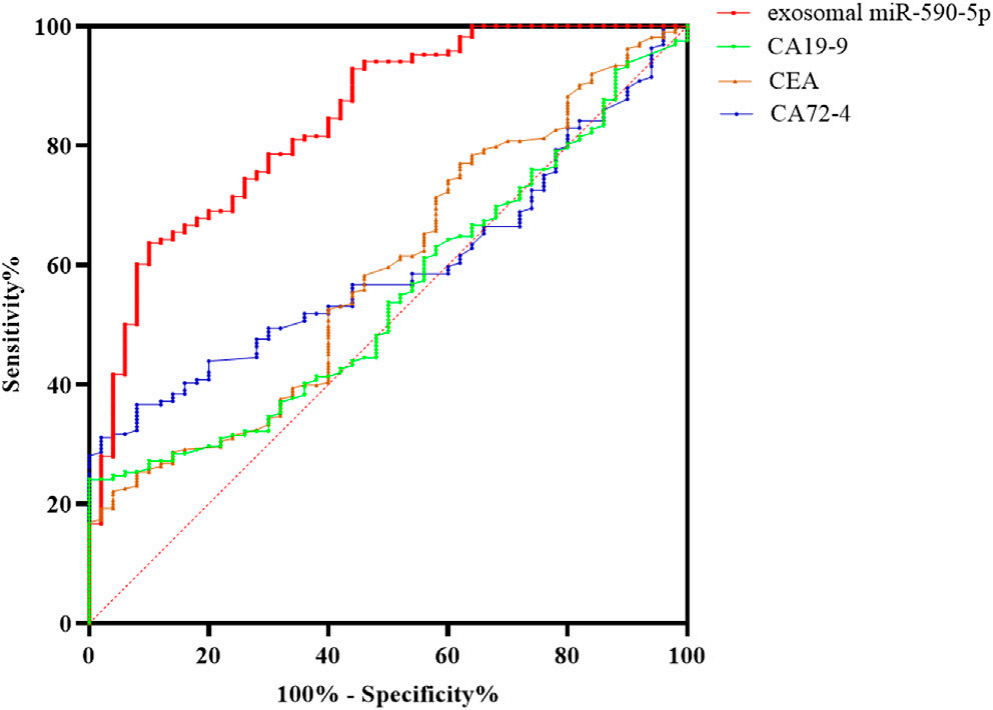
ROC curve analysis of serum exosomal miR-590-5p to discriminate gastric cancer with exosomal miR-590-5p and different tumor biomarkers from healthy controls. The results show that the exosomal miR-590-5p produces 0.810 below the curve, with 63.7% sensitivity and 86.0% specificity. The sensitivity and accuracy of the diagnosis of GC by serum exosomal miR-590-5p were significantly higher than those of any serum tumor markers.

ROC curve was plotted according to serum exosomal miR-590-5p expression. As shown in Figure 3, the serum exosomal miR-590-5p was worth distinguishing GC patients from healthy controls. As soon as the cutoff value reached 3.47, the ROC showed that serum exosomal miR-590-5p revealed a good classifier with an AUC of 0.810 (95% CI = 0.751–0.860) exhibiting a sensitivity of 63.7% and specificity of 86.0%. According to our results serum exosomal miR-590-5p expression may be a noninvasive diagnostic biomarker of gastric cancer.

### The Relationship Between the Expression of miR-590-5p and Clinicopathological Factors in Gastric Cancer Patients

In order to better understand the potential role of the exosomal miR-590-5p in the development of gastric cancer, the potential association of serum exosomal miR-590-5p levels with various clinicopathological features of GC was analyzed. The 168 patients with stomach cancer were divided into high expression groups and weak expression groups with the expression value of the intermediate exosomal miR-590-5p as a tangent point. Table 1 summarizes the relationship between the level of expression of the exosomal miR-590-5p and the clinical characteristics of gastric cancer. The results showed that the serum level of exosomal miR-590-5p in patients with gastric cancer was strongly related to the TNM phase (*p* = 0.008), the depth of infiltration, and the expression level of ki-67 (*p* <0.001). However, there is no significant correlation between expression of exosomal miR-590-5p and other clinical pathological characteristics such as age, sex, tumor size, tumor part, intravenous infiltration, cell differentiation, lymph node transfer, *Helicobacter pylori*, and the expression level of her-2 (all at *p* > 0.05).

**TABLE 1:** Clinicopathological correlations of serum exosomal miR-590-5p expression in 168 gastric cancer (GC) patients.

Clinicopathologic factor	miR-590-5p expression		χ^2^ value	*p* value
	Low	High		
Age			2.386	0.122
<60 years	45	35		
≥60 years	39	49		
Gender			0.253	0.615
Male	60	57		
Female	24	27		
Tumor size			0.858	0.354
<5 cm	38	44		
≥5 cm	46	40		
Tumor site			2.425	0.489
Cardia	8	4		
Body	14	12		
Antrum	23	30		
More than two parts	39	38		
Venous invasion			0.858	0.354
Absent	38	44		
Present	46	40		
Differentiation			0.869	0.351
Poor	44	50		
Well/Moderate	40	34		
T stage			23.899	<0.001
T1 + T2	5	31		
T3 + T4	79	53		
N Stage			0.136	0.712
N0 + N1 + N2	42	36		
N3	42	48		
TNM stage			6.929	0.008
I + II	11	25		
III	73	59		
*Helicobacter pylori*				
+	44	38	0.858	0.354
−	40	46		
Ki-67				
<40%	36	51	5.364	0.021
≥40%	48	33		
Her-2				
+	39	40	0.167	0.683
−	45	44		

### Exosomal miR-590-5p Expression in Serum Is Related to Survival Rate of Gastric Cancer Patients

**Figure 4:**
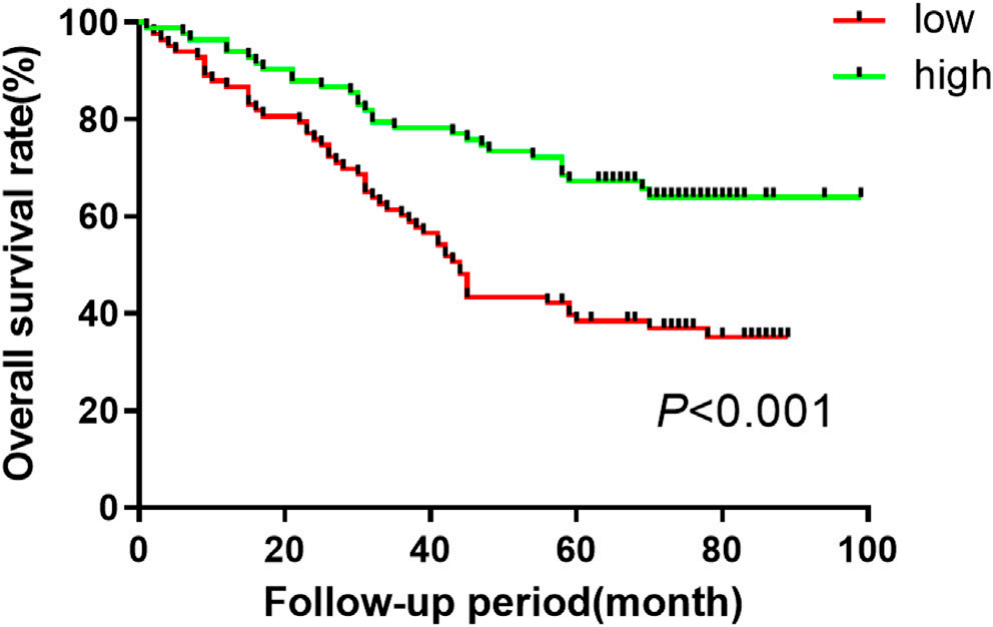
Kaplan-Meier curves of overall survival for GC patient based on serum miR-590-5p expression. Log-rank tests are used for relatively low and high survival rates of patients in the miR-590-5p expression group based on the median of the miR-590-5p expression.

In order to assess the precalculated value of exosomal miR-590-5p in patients with GC, patients were dichotomized into two groups of high or low expression level as previously mentioned. The log-rank test suggested that the high expression group appeared to show improvement of overall survival (26.2 months) compared to the low expression group (15.5 months for overall). Moreover, the results showed a significant association between low expression exosomal miR-590-5p and poor survival (*p* < 0.001, Figure 4), indicating that the exosomal miR-590-5p can be used as a potential prognosis indicator for patients with stomach cancer. In addition, as Table 2 shows, analysis of the Cox proportional risk regression model, a single variable, shows that the OS is strongly related to tumor size (*p* = 0.003), invasion depth (*p* < 0.001), clinical stage (*p* = 0.006), and exosomal miR-590-5p level (*p* < 0.001). In multivariate analysis, expression level of exosomal miR-590-5p remained significant (*p* = 0.013). The other independent prognostic factor was the cancer invasion depth (*p* = 0.005).

**TABLE 2:** Univariate and multivariate analysis of prognostic parameters in patients with GC by Cox regression analysis.

Variables	Univariate analysis		Multivariate analysis	
	HR (95% CI)	*p* value	HR (95% CI)	*p* value
Age				
≥60 vs. <60 years	1.01 (0.66–1.56)	0.960		
Gender				
Male vs. female	0.90 (0.56–1.46)	0.669		
Tumor size			1.296	
≥5 cm vs. <5 cm	1.94 (1.26–3.00)	0.003	(0.805–2.087)	0.285
Venous invasion				
Present vs. absent	1.36 (0.88–2.10)	0.168		
Differentiation				
Poor vs. well/Moderate	1.34 (0.87–2.06)	0.194		
Invasion depth			3.569	
T3+T4 vs. T1+T2	4.73 (1.71–4.52)	<0.001	(1.466–8.692)	0.005
Lymph node status			1.512	
N3 vs. N0+N1+N2	1.36 (0.88–2.10)	0.167	(0.958–2.388)	0.076
TNM stage				
Ⅲ vs. Ⅰ+Ⅱ	2.46 (1.24–3.34)	0.006		
miR-590-5p expression			0.558	
Low vs. high	2.31 (1.51–3.61)	<0.001	(0.351–0.886)	0.013

Abbreviations: CI = confidence interval; HR = relative risk.

### Improving the Level of miR-590-5p Expression by Mimics

**FIGURE 5:**
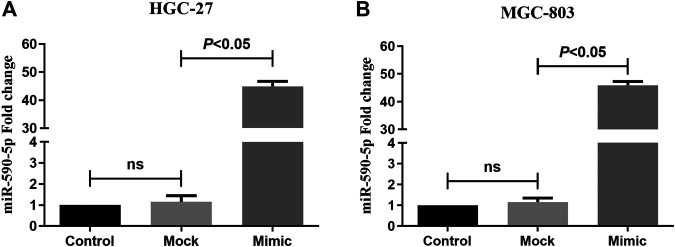
The expression of miR-590-5p in HGC-27 and MGC-803 cells. Compared to mock and control, cells that were transformed with the miR-590-5p simulation showed a higher miR-590-5p expression. ns *p* > 0.05.

To monitor the expression of miR-590-5p in gastric cancer cells, miR-590-5p mocks are delivered to HGC-27 and MGC-803 cells. 24 h after the infection, the expression level of miR-590-5p was detected through qRT-PCR. Expression of miR-590-5p among cells transmitted by miR-590-5p mimic increased by about 45 times (Figure 5). These results were used as the basis for determining subsequent experiments.

### MiR-590-5p Inhibited Gastric Cancer Cells Migration and Invasion *In Vitro*

**FIGURE 6:**
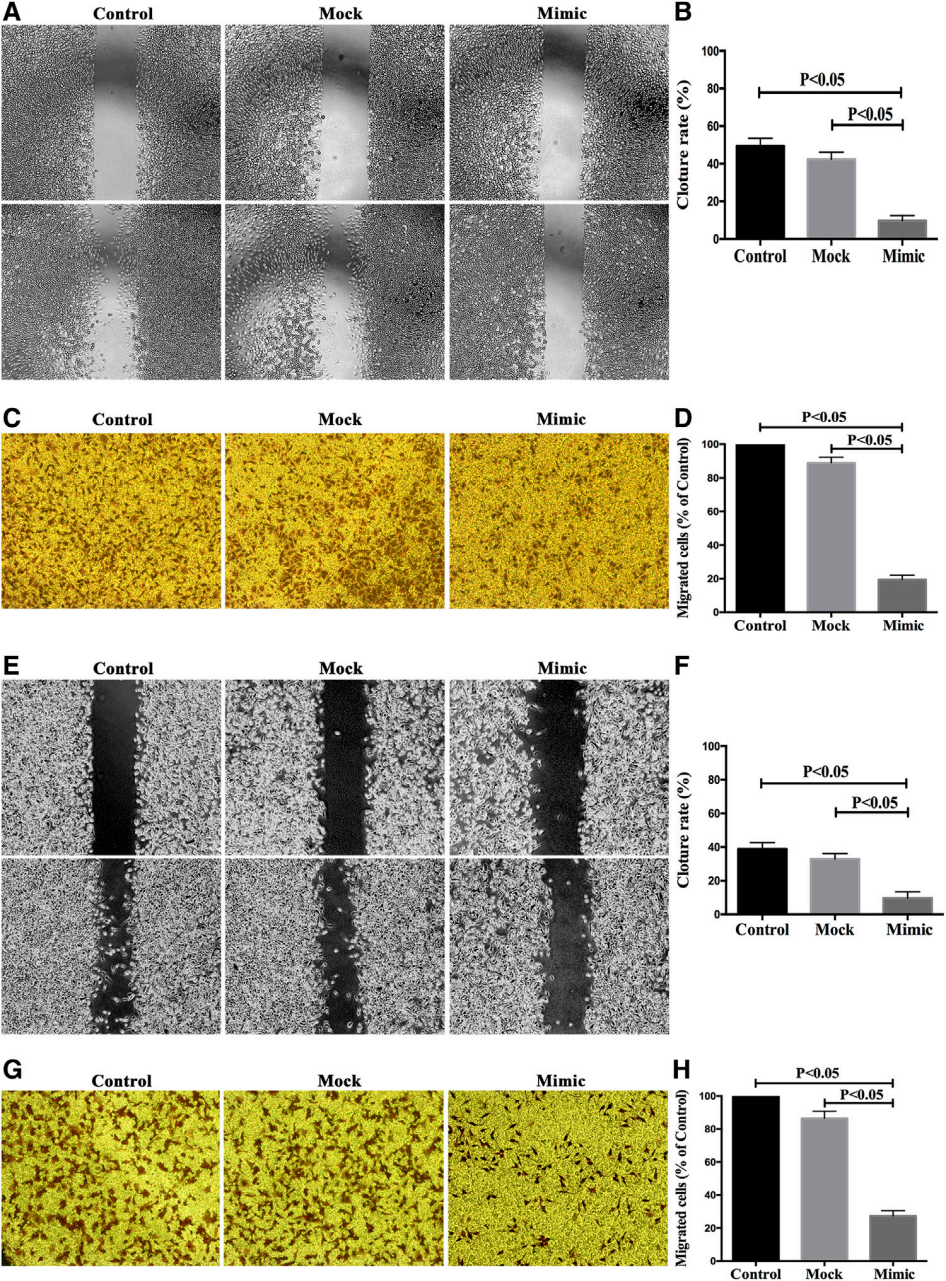
The overexpression of miR-590-5p inhibits *in vitro* the migration and intrusion of MGC-803 and HGC-27 cells. Effect of miR-590-5p on cell mobility and attack capability of MGC-803 and HGC-27 cells. The number of cells was observed under an inverted microscope 100 times larger and counted in each field. Three independent experiments were conducted. The results are indicated by the average standard deviation.

In order to study the role of miR-590-5p in gastric cancer metastasis, the scratch experiment and transwell experiments were performed to see if miR-590-5p is associated with the movement and encroachment of gastric cancer cells. As shown in Figure 6, the migration and attack capacity of the MGC-803 and HGC-27 cells was significantly reduced after the conversion of the miR-590-5p mimics. These observations show that high expression of miR-590-5p may play an important role in stemming the spread and invasion of gastric cancer cells.

## Discussion

As it is reported that miRNA can be detected in the serum and express itself regularly at room temperature and in many freezing cycles, it is widely accepted that circular miRNAs can be used as new noninvasive biological markers of cancer, prostate cancer and colorectal cell carcinoma (Wang et al., 2015; Fabris et al., 2016; Nagata et al., 2016; Schou et al., 2016). More than 100 miRNAs have been found to be aberrantly expressed in GC. However, there is still no description of exosomal miRNAs in GC; here we reveal that high exosomal miR-590-5p expression was significantly better for prognosis of GC. By targeting mRNA translation, microRNA can regulate cell proliferation, invasion, and migration. Due to the diversity of miRNAs and the complexity of the regulatory mechanism, it is difficult to determine whether a particular miRNA is carcinogenic or tumor suppressive (Svoronos et al., 2016). MiR-590-5p is not unique to many types of substances but is also overexpressed in the SW480 cell line and was found to inhibit SMAD3 protein expression (Jafarzadeh and Soltani, 2016). Jiang and others found that the decline in miR-590-5p resulted in an increase in TGF-beta RII and inhibited the proliferation and invasion of HepG2 cells (Jiang et al., 2012). Moreover, it has been shown that miR-590-5p can exert oncogenic activity in cervical carcinoma by targeting the CHL1 gene (Chu et al., 2014). However, in this study, the level of serum expression of miR-590-5p in patients with gastric cancer is significantly lower than that of the healthy control group. This inconsistency may be due to the differences in sample origin and the tumor clinicopathological characteristics. Moreover, the advanced level of miR-590-5p is also significantly lower than in the early stages, suggesting that exosomal miR-590-5p could be a promising biological marker for early gastric cancers, and yet, further validation is required to support this proposal.

ROC curves are widely used to assess diagnostic performance. In the current data, we compare serum exosomal miR-590-5p with traditional tumor markers CA72-4, CEA, and CA19-9 in serum, in the diagnosis of GC. Yet traditional serum tumor markers show a poor sensitivity and specificity; serum exosomal miR-590-5p achieved good diagnostic efficacy by distinguishing GC patients with exosomal miR-590-5p and different tumor markers from those with health control, with an AUC of 0.810 (sensitivity = 63.7%, specificity = 86%). Furthermore, it showed statistical significance with the clinical stage of GC. From reviewing and analyzing previous studies to our findings, serum exosomal miR-590-5p indicated a better sensitivity and specificity than serum tumor biomarkers which had reportedly low specificities and sensitivities (Schneider and Schulze, 2003; Yang et al., 2016); in addition, we were surprised to observe a negative correlation of exosomal miR-590-5p levels with increased ki-67 protein levels which is recognized to reflect the proliferation of tumor cells. However, this miRNA has no statistically significant correlation with *Helicobacter pylori* and her-2 protein levels which are independent risk factors affecting the prognosis of GC. The Kaplan-Meier analysis and Cox’s multivariate regression analysis showed that the low level of exosomal miR-590-5p reflected a much less favorable prognosis (*p* < 0.001). This suggests that the serum exosomal miR-590-5p might be a potential marker of poor prognosis in GC. Although lymph node status revealed a trend association without statistical significance in univariate analysis, after examining the depth of immersion, the level of serum exosomal miR-590-5p is significant as an independent prognostic factor through a Cox multivariate regression analysis, pathological grade, and lymph node status.

Given that the low serum level of miR-590-5p in patients with gastric cancer is related to the depth of infiltration, we further study the role of miR-590-5p in the cell transfer of human gastric cancer. Compared to the control group and the simulated group, after transfer with the miR-590-5p simulation, MGC-803 and HGC-27 cells were detected using the retroviral polymerase chain reaction. MiR-590-5p overexpression correlated with the decreased migration and invasiveness of MGC-803 cells and HGC-27 cells, indicating that it possessed a cancer suppressing role in GC. It is necessary to further explore the biological mechanism of miR-590-5p. Some research reports have shown miR-590-5p affects tumor cell epithelial-mesenchymal-transition (EMT), which plays an important role in tumor progression, and some related marker proteins such as β-catenin, N-cadherin, and Snail1 have changed (Jin et al., 2018; Khandelwal et al., 2019). We need to further study the role of miR-590-5p on the target genes of GC cells, which provides a new way for us to study the diagnosis, prognosis, and gene therapy of GC. We acknowledge that the study may have been more persuasive if larger samples had been used in the groups. Furthermore, the exact mechanism by which cancer cells secreted exosome-containing specific miRNAs has not been elucidated in detail. In order to clarify the biological mechanism of serum exogen miR-590-5p for patients with gastric cancer, further research on this topic is needed.

## Conclusion

The study showed that serum exosomal miR-590-5p might be a potential biological marker for the early detection of GC. Its downregulation may be related to poor prognosis in GC, which suggests that exosomal miR-590-5p could serve as promising biomarkers of further risk analysis for GC. This hopeful result has led to further research into the ambiguous mechanism of exosomal miR-590-5p as an intercellular messenger to regulate the invasion and transfer of gastric cancer.

## Data Availability

The original contributions presented in the study are included in the article/Supplementary Material; further inquiries can be directed to the corresponding author.
